# Empathy, access, and delays: determinants of oncology care satisfaction in Polish academic and nonacademic hospitals

**DOI:** 10.1093/oncolo/oyag046

**Published:** 2026-08-07

**Authors:** Natalia Sauer, Oskar Kozłowski, Dorota Bęben, Monika Birska, Sara Janczak, Anna Wiela-Hojeńska, Piotr Giedziun, Jacek Calik

**Affiliations:** Department of Clinical Pharmacology, Faculty of Pharmacy, Wroclaw Medical University, Wroclaw, 50-556, Poland; Faculty of Medicine, Wroclaw Medical University, Wroclaw, 50-367, Poland; Department of Basic Medical Sciences, Faculty of Pharmacy, Wroclaw Medical University, Wroclaw, 50-556, Poland; Department of Basic Medical Sciences, Faculty of Pharmacy, Wroclaw Medical University, Wroclaw, 50-556, Poland; Faculty of Medicine, Wroclaw Medical University, Wroclaw, 50-367, Poland; Department of Clinical Pharmacology, Faculty of Pharmacy, Wroclaw Medical University, Wroclaw, 50-556, Poland; Department of Artificial Intelligence, Wroclaw University of Science and Technology, Wroclaw 50-370, Poland; Division of Histology and Embryology, Department of Human Morphology and Embryology, Faculty of Medicine, Wroclaw Medical University, Wroclaw 50-368, Poland

**Keywords:** oncology care, specialist access, quality of care, oncology healthcare systems, patient well-being, cancer

## Abstract

**Background:**

Patient satisfaction is a critical measure of healthcare quality, particularly in oncology, where treatment outcomes are deeply intertwined with patients’ emotional and psychological experiences. This study assessed satisfaction levels among oncology patients treated in academic and nonacademic hospitals in Poland, identifying key determinants of satisfaction and disparities between these settings.

**Materials and Methods:**

We conducted a cross-sectional study of 546 oncology patients using a structured survey that assessed demographic, clinical, and experiential variables. Comparative and regression analyses evaluated the impact of factors such as staff empathy, specialist accessibility, and treatment delays on satisfaction. Subgroup analyses explored demographic differences, including age and gender, and their influence on patient experiences.

**Results:**

Patients treated in academic hospitals reported significantly higher satisfaction compared to those in nonacademic settings (*P* < .05). Key predictors of satisfaction included respect and empathy (coefficient = 0.338, *P* < .001), access to specialists (coefficient = 0.170, *P* < 0.001), and shorter treatment delays (coefficient = −0.426, *P* < .001). Younger patients and women exhibited greater emotional distress and financial concerns, with the <30-year age group reporting the highest financial fear (61.5%). Treatment delays in nonacademic hospitals were associated with a 42.6% reduction in the likelihood of satisfaction per additional month (*P* < .001).

**Conclusions:**

This study highlights the systemic and demographic disparities in oncology care satisfaction in Poland, with academic hospitals demonstrating superior outcomes. Addressing treatment delays, enhancing access to specialists, and prioritizing empathy in patient interactions are critical to improving satisfaction. Targeted support for younger patients and women, including psychological and financial interventions, is urgently needed to mitigate the disproportionate burden they face. These findings provide actionable insights to bridge gaps in oncology care quality and equity.

Implications for Practice This study identifies key priorities for improving oncology care delivery. Empathy, timely access to specialists, and minimizing treatment delays are central to patient satisfaction and directly influence treatment adherence. Elevated anxiety and reduced trust in medical staff—particularly in nonacademic settings—may compromise compliance, while financial distress, especially among younger patients, underscores the need for integrated psychosocial and financial support. At the system level, observed disparities between academic and nonacademic hospitals warrant caution in the decentralization of oncology services. Expanding geographic access without parallel investment in staffing, communication standards, and patient support infrastructure risks exacerbating inequalities in care quality. Standardization of organizational and psychosocial care across institutions is therefore essential to ensure equitable, continuous, and patient-centered oncology care.

## Introduction

Oncology care requires not only precise medical interventions but also a nuanced understanding of patient experiences and expectations.^1^^,^^2^ Patient satisfaction, widely recognized as a key indicator of healthcare quality, reflects the effectiveness of treatment organization, clinical communication, and institutional performance.^3–5^ Beyond objective clinical outcomes, patients’ emotional and psychological experiences within the healthcare system play a decisive role in shaping their overall perception of care.^6^^,^^7^

Empathy constitutes a core component of patient-centered oncology care and has been consistently associated with improved clinical outcomes, enhanced self-efficacy, reduced psychological distress, and higher patient satisfaction.^7^ Despite its acknowledged importance, empathy remains inconsistently conceptualized and measured, often with limited incorporation of the patient perspective. Recent studies emphasize the need for more patient-centered frameworks, demonstrating that oncology patients prioritize clinician behaviors such as active listening, understanding, and attentiveness to their broader psychosocial needs.^8^^,^^9^

Periods of heightened systemic strain have further underscored the importance of empathic communication and organizational resilience.^10^ Evidence indicates that patient satisfaction during such periods is strongly associated with clinicians’ ability to maintain transparency, accessibility, and emotional support despite organizational constraints. Moreover, persistent disparities in satisfaction related to demographic factors—including age, gender, and place of residence—highlight the need for tailored interventions to address specific patient vulnerabilities.^11^

High-quality communication, encompassing both verbal and nonverbal dimensions, represents a cornerstone of empathic oncology care.^12–14^ Patients consistently identify clear information provision, respect, and a compassionate professional attitude as fundamental to a supportive therapeutic relationship. Importantly, perceived communication quality significantly influences trust in physicians, a critical determinant of treatment engagement and continuity.^15^ In this context, clinician attributes such as accessibility and perceived competence extend traditional definitions of empathy, reflecting the distinctive demands of cancer care.^7^

Against this background, the present study examines patient satisfaction with oncology care in Polish hospitals, with particular attention to healthcare accessibility, communication clarity, and staff empathy. By comparing academic and nonacademic institutions, the study aims to identify systemic differences shaping patient experience. In addition, demographic characteristics (age, gender, education) and clinical variables (time since diagnosis, treatment duration, prior surgery) are analyzed alongside psychological and organizational challenges, including treatment delays, access to innovative therapies, and emotional distress related to cancer diagnosis and treatment.

## Methods

### Study design and participants

This cross-sectional study assessed patient satisfaction with medical care in the Clinical Oncology Department at University Clinical Hospital in Wrocław, Poland (Academic Hospital), and compared these findings with patient satisfaction data from oncology departments in other public hospitals nationwide. Data were collected both in person and via online forms distributed through cancer support groups. A total of 546 questionnaires were completed, with 163 collected in person and 384 online. Eligible participants were adults (≥18 years) undergoing chemotherapy, regardless of cancer type, stage, or prior surgical interventions. Both inpatient and outpatient oncology patients were included if they had received care for at least 1 day in the department or, for outpatients, if they had attended at least 1 therapeutic session. Patients admitted on the survey day were excluded. All participants provided informed consent, and the study was conducted in accordance with institutional guidelines. Table S1 describes the patients characteristics.

### Survey instrument

A custom-designed, structured questionnaire was developed to assess patient satisfaction with oncology care. The survey comprised 4 sections addressing: (1) sociodemographic and treatment characteristics; (2) satisfaction with treatment organization and quality of care, including access to specialists and perceived staff professionalism; (3) treatment-related concerns and psychological distress; and (4) additional treatment aspects, such as information-seeking behavior, secondary consultations, and perceived clarity of medical explanations.

Key constructs, including respect and empathy, specialist access, explanation clarity, and treatment-related fears, were assessed using patient-reported perceptions. Detailed definitions of individual domains and anxiety measures are provided in Supplementary Material 1.

The questionnaire underwent pilot testing in 15 oncology patients to assess clarity and comprehensibility, followed by a test–retest reliability assessment in 20 patients over a 7-day interval, demonstrating good temporal stability. Participants involved in pilot testing were excluded from the main study cohort. For patients treated outside the Wrocław Clinical Oncology Department, an open-ended item captured the treatment location.

### Data collection procedures

Data were collected between March and November 2024. In the Clinical Oncology Department in Wrocław, surveys were administered in person by a trained survey administrator. Participants completed the questionnaire anonymously, and survey completion was estimated to take approximately 10 minutes. To gather data from other public oncology departments, the questionnaire was distributed through patient support groups on Facebook identified as relevant to oncology patients. Patients were informed that participation was voluntary, and the survey’s anonymity was emphasized to encourage candid responses.

### Sample size and data management

The target sample size was 546 completed surveys to ensure sufficient statistical power and representation of patients from both the Wrocław Clinical Oncology Department and other public hospitals. Surveys were monitored to achieve balanced contributions from both groups. Data were de-identified, and responses were stored securely to protect patient confidentiality. Descriptive and inferential analyses were conducted to compare satisfaction levels across institutions, and subgroup analyses were performed based on demographic and treatment characteristics.

### Statistical analysis

The statistical analysis was conducted using Python libraries to ensure precision and reproducibility. Data preprocessing and management were performed with pandas (v2.2.2), while numerical computations utilized NumPy (v1.26.4). Descriptive statistics, including means, medians, and standard deviations for continuous variables, and frequencies for categorical variables, were calculated. Inferential statistics involved χ^2^ tests for associations between categorical variables and Spearman and Pearson correlation analyses to assess relationships between selected variables.

Multivariable regression models, including linear and ordinal logistic regression, were implemented using statsmodels (v0.14.4) to investigate predictors of treatment satisfaction and care quality while controlling for potential confounding variables. Residual diagnostics were conducted to verify that model assumptions were met. Confounding variables were identified based on theoretical considerations and empirical evidence, ensuring that their effects were appropriately adjusted for in the models.

Visualizations, such as boxplots, scatter plots, and heatmaps, were generated using Matplotlib (v3.8.0) and Seaborn (v0.13.2) to enhance the interpretability of the results. Satisfaction scores and treatment factors were analyzed with ordinal encoding using sklearn (v1.3.0). Statistical significance was set at *P* < .05, and all analyses were conducted in Google Colaboratory to maintain a consistent computational environment.

## Results

### Academic hospitals show higher patient satisfaction and lower fear levels compared to nonacademic settings

Fear, anxiety, and satisfaction levels differed between patients treated in academic and nonacademic hospitals (Figure S1, see online supplementary material for a color version of this figure).

Initial fear was the most frequently reported concern in both settings, reported by 64.8% of patients in academic hospitals and 75.0% in nonacademic hospitals (*P* < .05). Pain fear was reported by 39.2% of patients in academic hospitals, while financial fear affected 35.2% of this group. Side effect fear and waiting period anxiety were reported by approximately 20% of patients. No significant differences between hospital types were observed for pain fear or side effect Fear (*P* > .05).

Patients treated in academic hospitals reported higher satisfaction across multiple care domains (Figure S1B, see online supplementary material for a color version of this figure). Respect and empathy and staff care received the highest ratings, with over 50% of respondents in academic hospitals reporting strong agreement, compared with approximately 44% in nonacademic hospitals. Satisfaction with visit access was lower in both settings, with only 29.5% of patients in nonacademic hospitals reporting satisfaction, compared with approximately 36% in academic hospitals.

Multivariable regression analysis identified respect and empathy and information provided as significant predictors of patient satisfaction in both hospital types (*P* < .001). The model explained 37% of the variance in satisfaction with care (R^2^ = 0.37). Visit access satisfaction remained significantly lower in nonacademic hospitals compared with academic hospitals (*P* < .05).

### Younger patients report significantly higher financial fears, especially in academic hospitals

**Figure 1.**
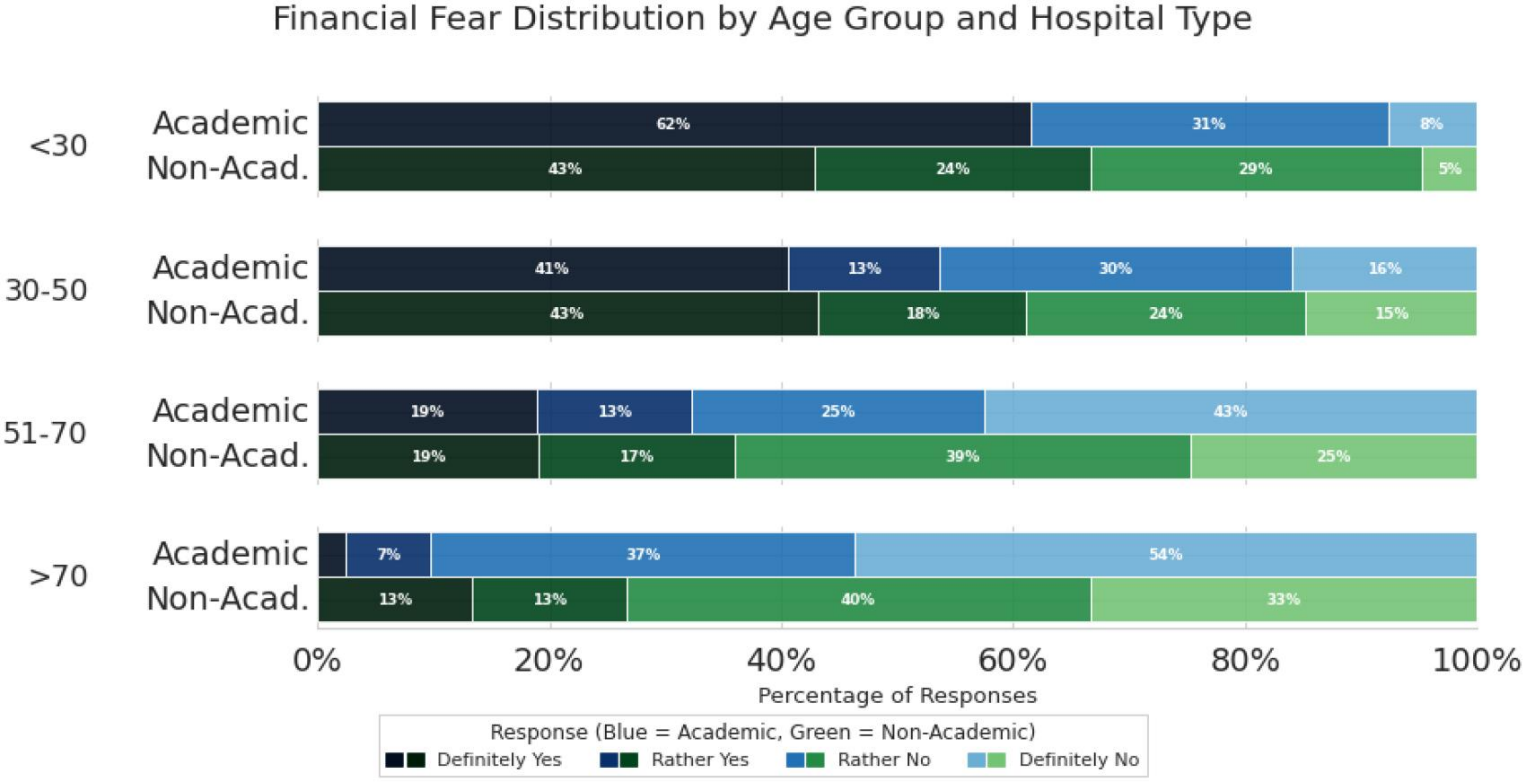
Distribution of financial fear across age groups in academic and nonacademic hospitals. The bars represent the percentage of individuals in each age group expressing varying levels of financial fear, ranging from “Definitely Yes” to “Definitely No.”

Significant differences in financial fear were observed across age groups and hospital types (Figure 1). Patients <30 years of age were significantly more likely to report strong financial fear than those >70 years of age, who were 94.3% less likely to report a “Definitely Yes” response.

In academic hospitals, strong financial fear (“Definitely Yes”) was reported by 61.5% of patients <30 years of age, compared with 18.9% among those aged 51-70 and 7.3% among patients >70 years of age. Conversely, the absence of financial fear (“Definitely No”) was reported by 53.7% of patients >70 years of age, compared with 7.7% in the youngest group.

In nonacademic hospitals, a similar age-related distribution was observed, although with lower levels of extreme concern. Strong financial fear was reported by 42.9% of patients <30, decreasing to 19.1% among those aged 51-70 and 13.3% among patients >70 years of age. The proportion reporting no financial fear (“Definitely No”) was highest among patients >70 years of age (33.3%) and lowest among patients >30 years of age (4.8%).

Multivariable regression analysis identified female gender, fear of side effects, the presence of family or friends during visits, and direct financial concerns as significant predictors of financial fear. Age group, education level, chronic pain, time since diagnosis, and treatment duration were not significant contributors in the adjusted model.

### Longer treatment delays lead to lower satisfaction, with nonacademic hospitals most affected

**Figure 2.**
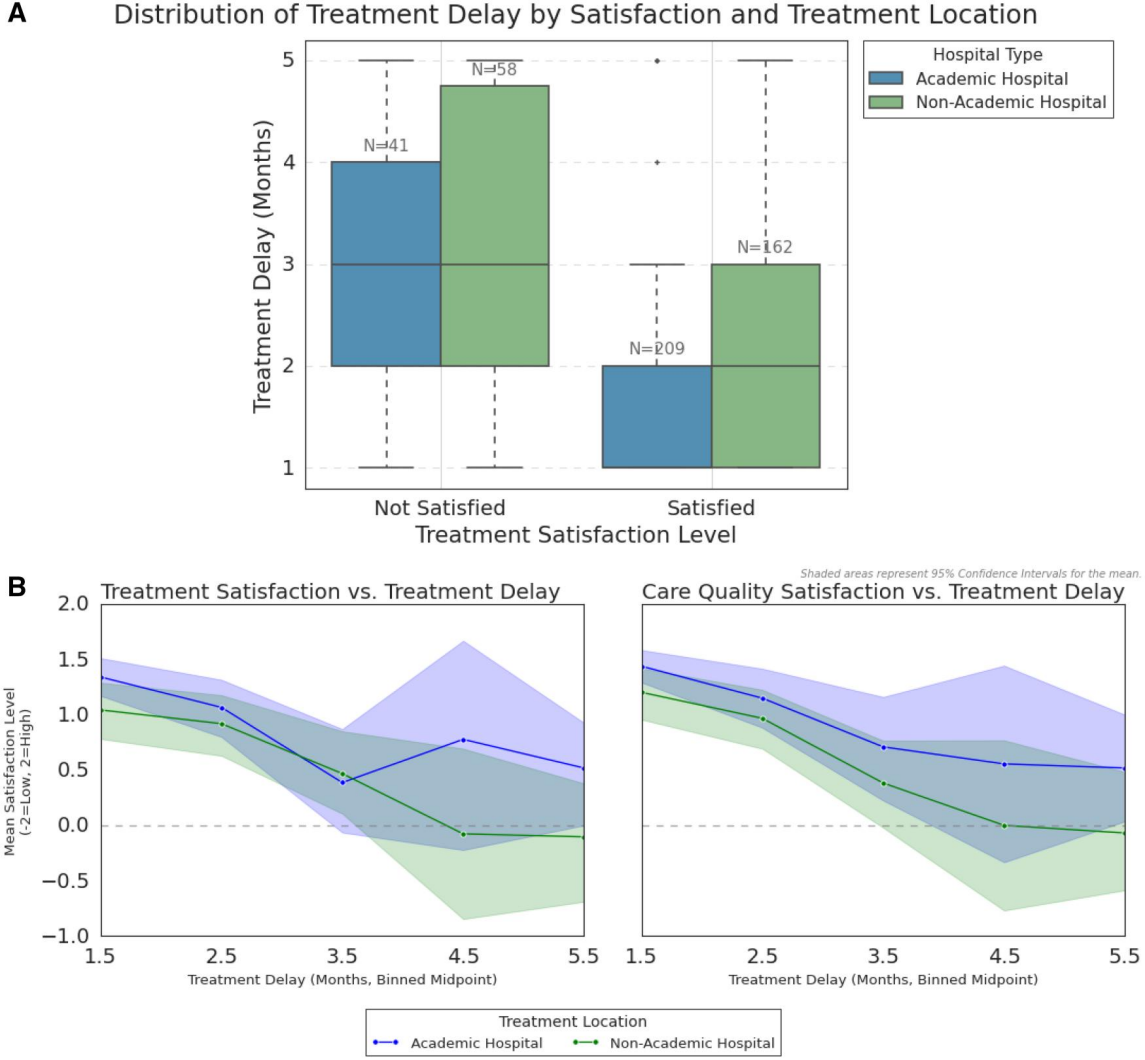
(A) Comparison of treatment delays and patient satisfaction with treatment progress, stratified by treatment location (academic vs nonacademic hospitals). The boxplots depict treatment delays (in months) for patients who reported being satisfied (“Satisfied”) or not satisfied (“Not Satisfied”) with their treatment. (B) Trends in satisfaction levels as treatment delays increase, stratified by treatment location. The left panel shows the decline in treatment satisfaction with increasing treatment delays, while the right panel illustrates the decrease in care quality satisfaction. Satisfaction levels are represented on a scale where higher values indicate greater satisfaction.

Treatment delays and satisfaction levels differed significantly between academic and nonacademic hospitals (Figure 2).

Among patients satisfied with treatment, the mean delay was 1.8 months (SD = 0.5) in academic hospitals and 2.4 months (SD = 0.7) in nonacademic hospitals. Among patients not satisfied with treatment, delays increased to 3.5 months (SD = 0.9) and 4.3 months (SD = 1.1), respectively. Differences in treatment delays between hospital types were statistically significant for both satisfied patients (t = 3.21, *P* = .002) and dissatisfied patients (t = 2.67, *P* = .008).

Logistic regression analysis demonstrated that each additional month of treatment delay was associated with a 42.6% reduction in the likelihood of treatment satisfaction (coefficient = −0.426, *P* < .001). Treatment in nonacademic hospitals was independently associated with lower odds of satisfaction compared with academic hospitals (coefficient = −0.462, *P* = .009).

Satisfaction declined progressively with increasing treatment delays in both settings (Figure 2B). The decline was more gradual in academic hospitals, whereas satisfaction levels in nonacademic hospitals approached their lowest values after a delay of 5 months. Similar patterns were observed for care quality satisfaction. Each additional month of delay significantly reduced care quality satisfaction (coefficient = −0.477, *P* < .001), and treatment in nonacademic hospitals was associated with substantially lower odds of care quality satisfaction (coefficient = −0.635, *P* < .001).

### Respect, empathy, specialist access, and clear communication drive care quality satisfaction

**Figure 3.**
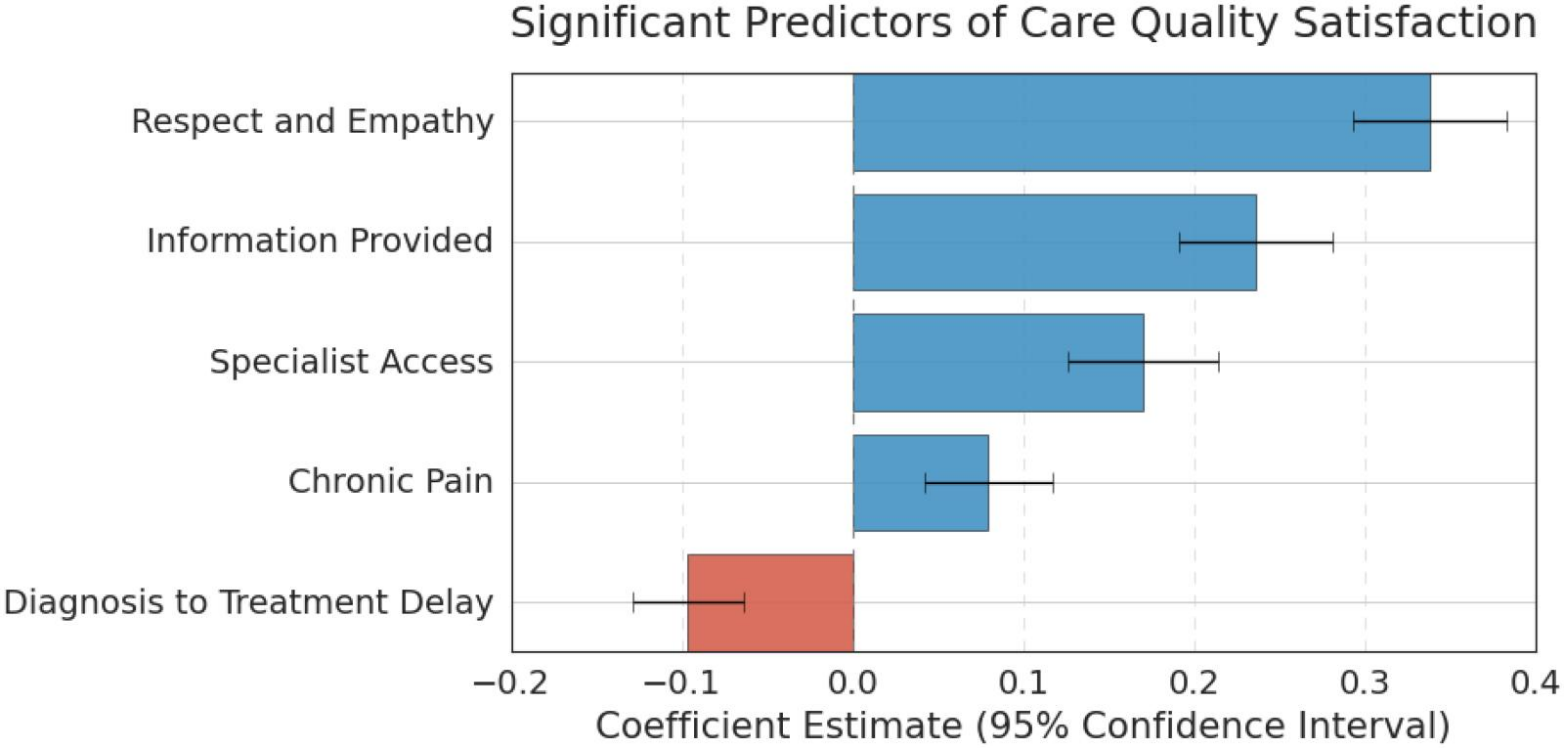
Significant predictors of care quality satisfaction identified through multivariable regression analysis. Bars represent coefficient estimates for each predictor, with error bars indicating 95% confidence intervals.

In order to identify and better understand the key factors influencing patient satisfaction with care quality, a comprehensive multivariable regression analysis was conducted, focusing on both patient-reported experiences and clinical variables to inform targeted improvements in healthcare delivery (Figure 3).

Multivariable regression analysis identified several significant predictors of care quality satisfaction (Figure 3). Respect and empathy showed the strongest positive association (coefficient = 0.338, *P* < .001), followed by information provided about treatment (coefficient = 0.236, *P* < .001). Access to specialists was also positively associated with care quality satisfaction (coefficient = 0.170, *P* < .001).

Diagnosis-to-treatment delay was negatively associated with care quality satisfaction (coefficient = −0.097, *P* = .003). Chronic pain showed a weak positive association (coefficient = 0.079, *P* = .035).

Several variables were not significant predictors of care quality satisfaction, including waiting period anxiety (*P* = .493), treatment location (*P* = .947), access to innovative or alternative treatments (*P* > .4), education level, age group, side effect concerns, pain, financial fear, treatment duration (all *P* > .2), and the presence of support during treatment (*P* = .475).

### Emotional comfort with doctors protects against initial treatment anxiety

**Figure 4.**
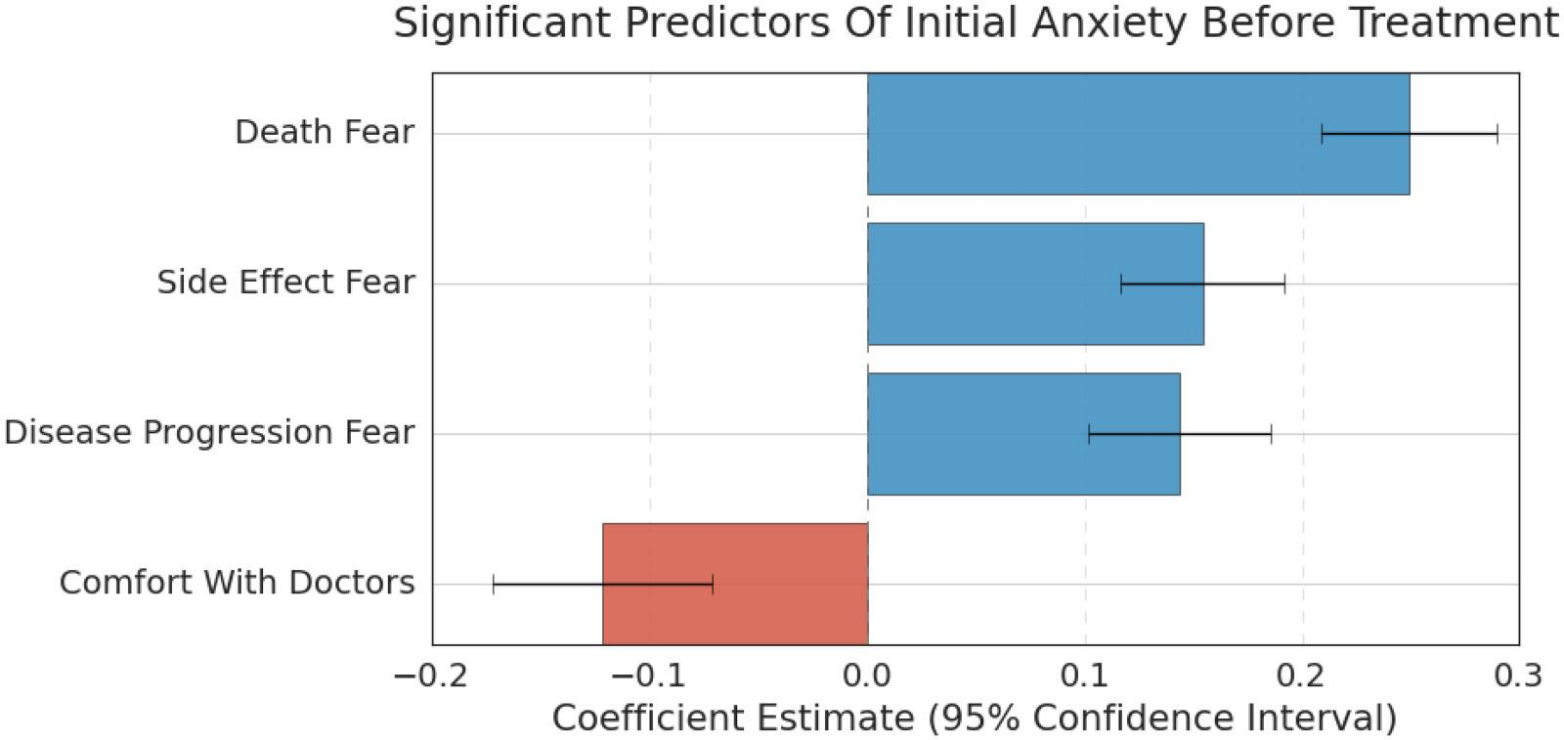
Significant predictors of initial anxiety before treatment, as identified through multivariable regression analysis. Bars represent the coefficient estimates for each predictor, with error bars indicating 95% confidence intervals. Positive coefficients (eg, death fear, side effect fear, and disease progression fear) indicate predictors associated with higher levels of initial anxiety, while the negative coefficient for comfort with doctors reflects its protective effect against anxiety. Non-significant predictors, such as financial fear, support presence, and empathy from medical staff, are not displayed in the figure.

Multivariable regression analysis was performed to identify factors associated with initial anxiety before treatment, incorporating patient-reported fears, emotional responses, and clinical interaction variables (Figure 4).

Several variables were significantly associated with higher levels of initial anxiety. Death fear emerged as the strongest predictor (coefficient = 0.251, *P* < .001), followed by fear of side effects (coefficient = 0.156, *P* < .001) and fear of disease progression (coefficient = 0.145, *P* = .001). These factors were consistently associated with increased anxiety prior to treatment initiation.

In contrast, emotional comfort with doctors showed a significant protective effect against initial anxiety, with a negative association observed in the regression model (coefficient = −0.124, *P* = .015). Patients reporting greater comfort during interactions with physicians experienced lower levels of pretreatment anxiety.

Several variables were not significantly associated with initial anxiety. These included the presence of family or friends during visits (*P* = .276), financial fear (*P* = .214), and perceived respect and empathy from medical staff (*P* = .207). Other factors, such as loss of hope (*P* = .165), psychological deterioration, loneliness, chronic pain, and access to innovative treatments, were also not significant predictors (all *P* values > .2).

Overall, the results indicate that fears related to death, treatment side effects, and disease progression are key contributors to initial anxiety, whereas emotional comfort with doctors is associated with lower anxiety levels before treatment.

### Treatment satisfaction and communication clarity are key predictors of patient trust in doctors

**Figure 5.**
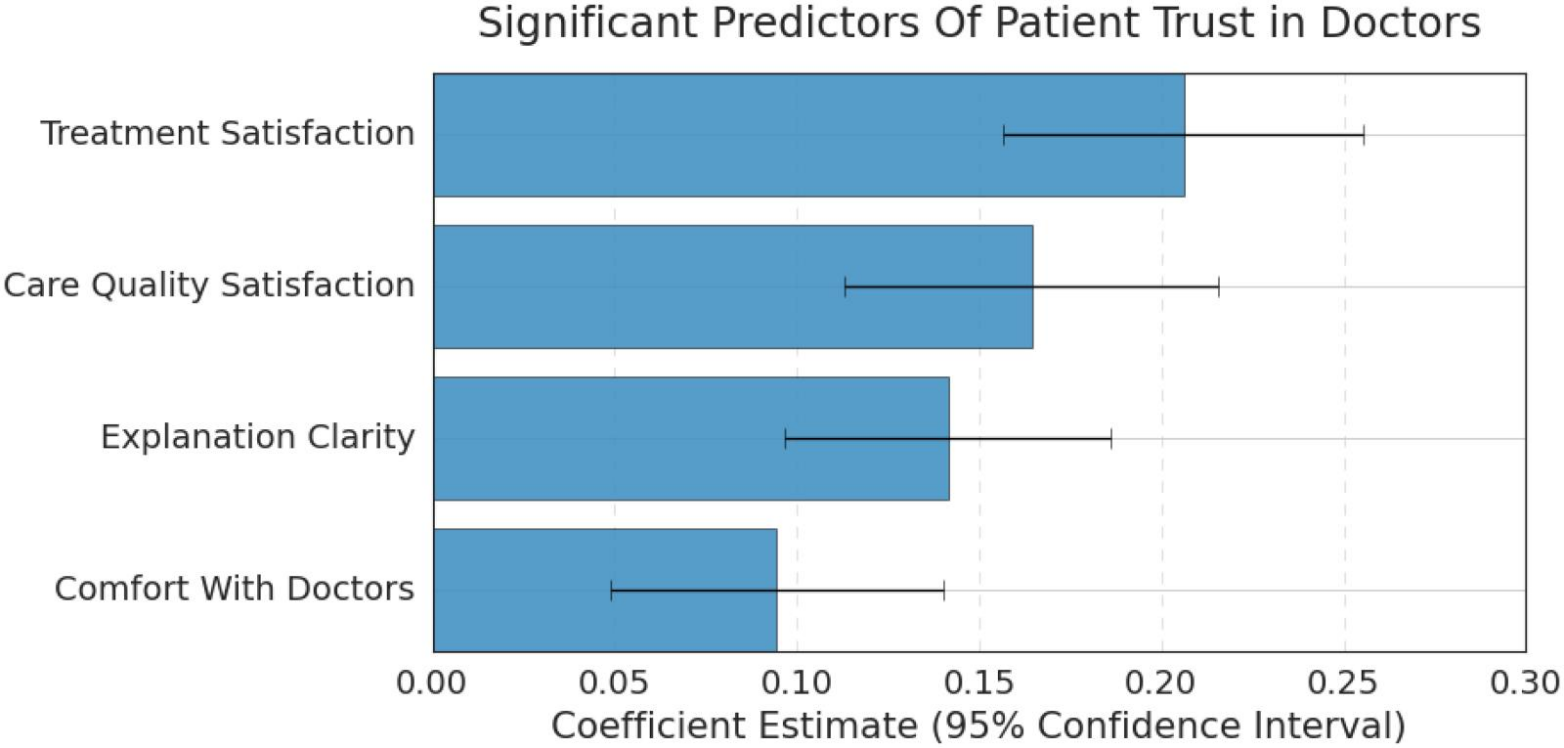
Significant predictors of patient trust in doctors identified through multivariable regression analysis. Bars represent coefficient estimates for each predictor, with error bars indicating 95% confidence intervals. Positive coefficients (treatment satisfaction, care quality satisfaction, explanation clarity, and comfort with doctors) indicate factors positively associated with higher trust in doctors. Non-significant variables, such as respect and empathy, information provided, and emotional factors, are not included in the figure.

Multivariable regression analysis was conducted to identify factors associated with patient trust in doctors, incorporating treatment satisfaction, perceived care quality, communication-related variables, and patient experiences (Figure 5).

The model explained a substantial proportion of the variance in trust in doctors, with an adjusted R^2^ of 0.462. The overall model was statistically significant (F = 24.71, *P* < .001), indicating strong explanatory power.

Treatment satisfaction emerged as the strongest predictor of trust (coefficient = 0.206, *P* < .001). Care quality satisfaction was also positively associated with trust in doctors (coefficient = 0.164, *P* < .001). Communication-related factors contributed independently, with explanation clarity showing a significant positive association (coefficient = 0.141, *P* < .001). Emotional comfort with doctors was likewise associated with higher trust levels (coefficient = 0.094, *P* = .015).

Several variables were not significantly associated with trust in doctors (*P* > .05). These included respect and empathy, information provided, emotional factors such as initial fear, loneliness, and psychological deterioration, as well as demographic characteristics (age and education level). External behaviors, including seeking second opinions or consulting online sources, and staff availability also showed no significant association with trust.

### Clarity of medical explanations is shaped by trust, empathy, and communication quality

**Figure 6.**
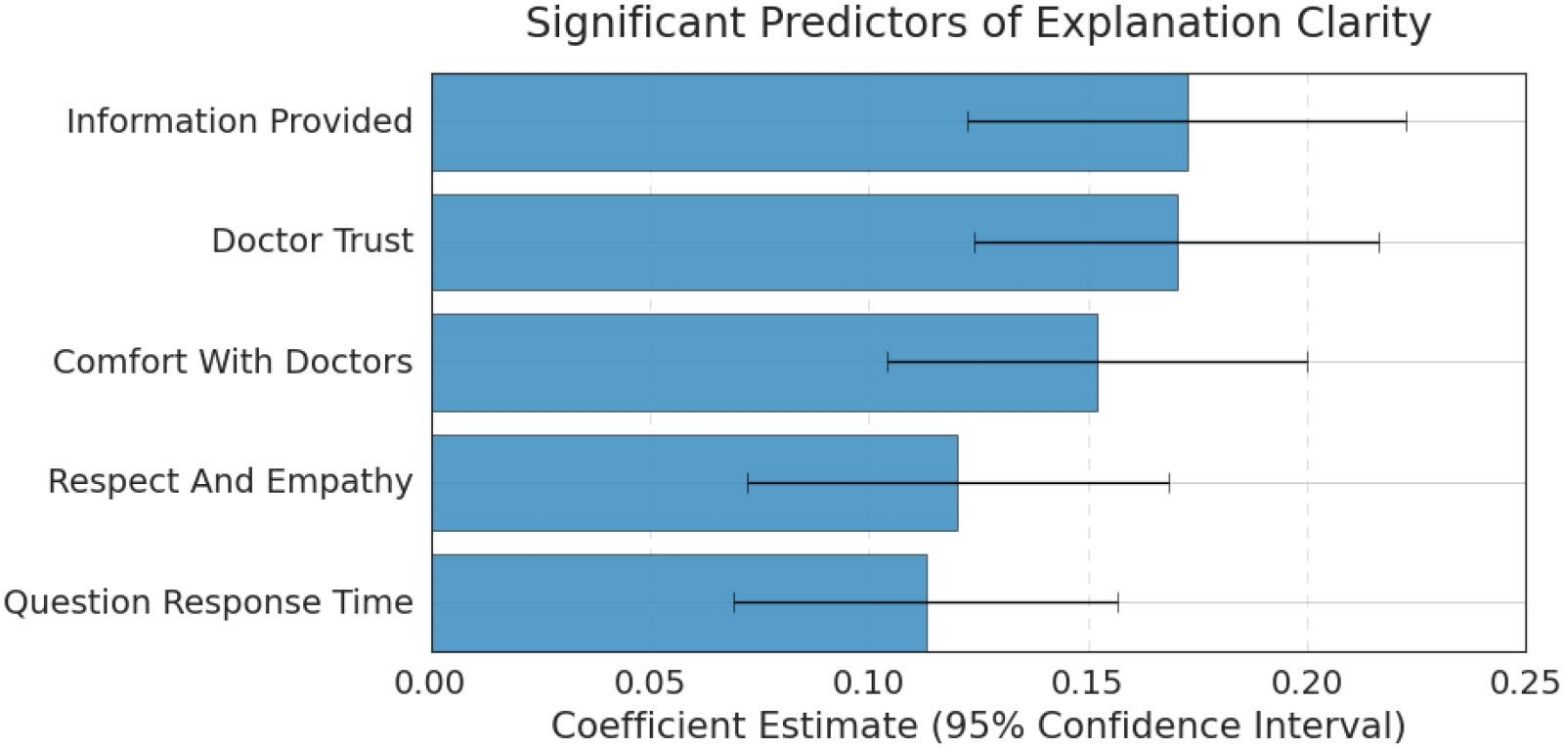
Significant predictors of explanation clarity identified through multivariable regression analysis. Bars represent coefficient estimates for each predictor, with error bars indicating 95% confidence intervals. Positive coefficients (information provided, respect and empathy, comfort with doctors, doctor trust, and question response time) indicate factors that significantly enhance explanation clarity. Non-significant variables, such as treatment duration, age group, support presence, and gender, are not included in the figure.

To investigate the factors that significantly influence patients’ understanding of medical explanations, we performed a multivariable regression analysis identifying key predictors of explanation clarity (Figure 6).

The model explained 49% of the variance in explanation clarity (R^2^ = 0.49), indicating strong explanatory power. Among the significant predictors, information provided was strongly associated with improved explanation clarity (*P* = .001). Respect and empathy (*P* = .013) and comfort with doctors (*P* = .002) were also positively associated with patients’ understanding of medical explanations, underscoring the role of interpersonal aspects of care.

Doctor trust emerged as the strongest predictor of explanation clarity (*P* < .001). In addition, question response time was significantly associated with explanation clarity (*P* = .010), indicating that timely responses to patient inquiries contribute to better comprehension.

Several variables were not significant predictors of explanation clarity, including treatment duration, age group, the presence of support, and gender (all *P* values > .05).

Overall, the findings indicate that explanation clarity is primarily influenced by communication-related and relational factors, including information provision, trust, empathy, and comfort with doctors, whereas demographic and treatment-related variables did not significantly affect patients’ understanding in this model.

### Emotional and procedural factors contribute to anxiety while awaiting treatment

**Figure 7.**
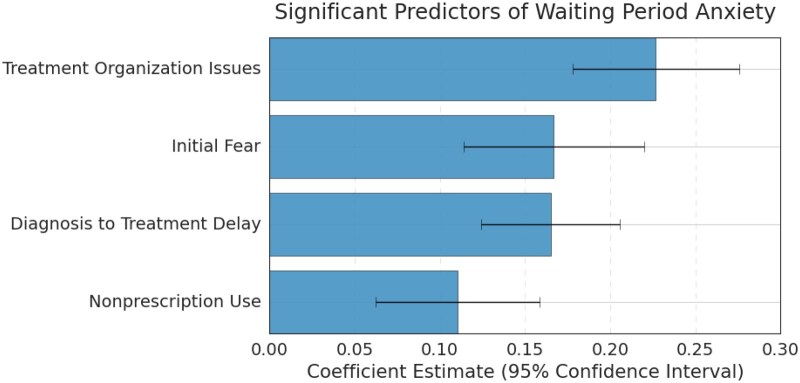
Significant predictors of waiting period anxiety identified through multivariable regression analysis. Bars represent coefficient estimates for each predictor, with error bars indicating 95% confidence intervals. Positive coefficients (treatment organization issues, initial fear, diagnosis-to-treatment delay, and nonprescription use) indicate factors that significantly increase waiting period anxiety. The figure does not include non-significant variables, such as care quality satisfaction, specialist access, doctor trust, and demographic factors (age and education).

We conducted analysis to identify the key factors influencing patient anxiety during the waiting period for treatment, focusing on care accessibility and organization, to highlight areas requiring improvement in the treatment process (Figure 7).

The model explained 17.4% of the variance in waiting period anxiety (R^2^ = 0.174), indicating a moderate explanatory capacity. Treatment organization issues emerged as the strongest predictor of anxiety (*P* < .001), with patients reporting organizational inefficiencies experiencing higher levels of anxiety while awaiting treatment.

Initial fear was also positively associated with waiting period anxiety (*P* = .002). In addition, diagnosis-to-treatment delay showed a significant positive association with anxiety (*P* < .001), indicating that longer waiting times were linked to increased distress. Non-prescription medication or supplement use was similarly associated with higher anxiety levels (*P* = .021).

Several variables were not significantly associated with waiting period anxiety (*P* > .05), including care quality satisfaction, specialist access, and trust in doctors, as well as demographic and clinical factors such as age group, gender, time since diagnosis, and education level.

Overall, the results indicate that organizational factors related to treatment planning and timing are primary drivers of anxiety during the waiting period, while emotional and behavioral factors, including initial fear and nonprescription use, contribute additionally to heightened anxiety.

## Discussion

This study highlights the crucial role of medical staff empathy, specialist availability, and treatment waiting times in shaping oncology patients’ satisfaction with care. These findings align with existing literature emphasizing the importance of effective communication and staff engagement in improving patient trust, psychological outcomes, and adherence to treatment protocols.^16^^,^^17^

A key finding is the significantly higher satisfaction reported by patients treated in academic hospitals, attributed to enhanced specialist access, multidisciplinary care, and shorter waiting times. International studies confirm that academic institutions offer superior organizational structures and cutting-edge therapies.^18–20^ In contrast, nonacademic hospitals, often affected by staffing shortages and resource constraints, struggle with delays, leading to lower patient trust and heightened distress.

The infrastructure in Polish academic hospitals often includes cutting-edge diagnostic tools and access to clinical trials. Patients treated in these facilities may benefit from personalized medicine approaches, including genetic testing for targeted therapies, which are less frequently available in nonacademic settings.^21^^,^^22^ Additionally, academic hospitals are typically at the forefront of adopting new technologies.

Demographic differences further shape patient experiences, with younger individuals and women reporting greater emotional distress and anxiety, consistent with previous studies.^23^^,^^24^ Younger patients, in particular, experience heightened financial insecurity, which may negatively impact treatment adherence. Despite the existence of the National Health Fund and public healthcare coverage in Poland, many young individuals remain concerned about financial stability when facing treatment interruptions, especially if they experience career breaks and have not yet accumulated savings.^25–30^ Poland’s healthcare system is structured around a national health insurance model administered by the National Health Fund (NFZ), which provides universal coverage for oncology services. Cancer diagnostics, hospitalizations, surgeries, chemotherapy, and radiotherapy are publicly financed and delivered free of charge at the point of care under this system. As a result, patients do not typically incur significant out-of-pocket expenses for standard cancer treatments, with the NFZ absorbing the vast majority of direct medical costs. Consequently, the main financial hardship reported by Polish cancer patients tends to arise from indirect costs—most notably loss of income due to work absence during treatment—rather than from medical bills. Indeed, evidence from European health systems indicates that in such third-party payer contexts, “financial toxicity” is driven more by lost earnings than by direct treatment expenses.^29^ Many patients, especially younger individuals early in their careers, express anxiety about their financial stability during prolonged therapy because of reduced income, even though their care itself is fully covered by public insurance. While direct medical costs are covered under Poland’s public healthcare system, our findings highlight that indirect economic pressures—particularly those stemming from income loss during treatment—remain a significant concern, especially among younger patients.

Beyond financial considerations, our study reveals notable disparities in the psychological experiences and satisfaction levels of oncology patients treated in academic versus nonacademic hospitals. Academic institutions appear to foster a more supportive treatment environment, effectively reducing patients’ initial fears and emotional burdens. In contrast, care provided in nonacademic hospitals is associated with elevated anxiety and lower satisfaction across multiple dimensions. For instance, the proportion of patients reporting high initial fear was substantially lower in academic hospitals (64.8%) than in nonacademic ones (75%). This difference may reflect greater access to pretreatment psychological counseling and more structured communication strategies in academic settings, both of which help manage expectations and emotional responses to diagnosis and treatment^31^. Conversely, patients treated in nonacademic hospitals may encounter systemic challenges such as limited psychological support, insufficient information delivery, and extended delays in treatment initiation, all of which contribute to increased distress.

These findings are consistent with international literature emphasizing the protective role of early psychosocial intervention in oncology care. Studies have shown that comprehensive pretreatment counseling can significantly reduce patient anxiety and enhance confidence in the therapeutic process.^32^ Our data suggest that the more robust infrastructure and multidisciplinary approach of academic hospitals contribute meaningfully to these improved psychosocial outcomes.

Among the strongest predictors of lower patient satisfaction in our study were treatment delays. Patients in academic hospitals experienced shorter wait times than those in nonacademic institutions; however, even within the former, anxiety related to waiting remained prevalent being reported by 41.2% of patients. This underscores the complexity of aligning system efficiency with patient expectations, even in well-resourced environments. Our results echo findings from European and Asian healthcare systems, where prolonged treatment delays have been consistently associated with elevated anxiety and decreased trust in healthcare providers.^17^^,^^33^^,^^34^

Beyond their effect on patient satisfaction, these findings have direct implications for treatment compliance and the structure of oncology service delivery. Heightened anxiety, lower trust in medical personnel, and insufficient psychosocial support—particularly prevalent in nonacademic settings—may contribute to reduced adherence to treatment protocols. Financial stressors, especially among younger patients, may further compromise compliance by discouraging follow-up visits or continuation of therapy during extended work absence. From a system perspective, the observed disparities raise important questions about the decentralization of cancer treatment. While expanding access beyond specialized centers may improve geographic availability, our data suggest that without adequate investment in staffing, communication standards, and patient support infrastructure, such delocalization risks exacerbating inequities in care quality. Ensuring uniform psychosocial and organizational standards across treatment settings will be essential to safeguard treatment continuity and equity in outcomes.

## Conclusions

This study highlights significant disparities in oncology care experiences between academic and nonacademic hospitals in Poland, emphasizing the critical roles of staff empathy, specialist accessibility, and treatment delays in shaping patient satisfaction. Patients treated in academic hospitals reported higher satisfaction levels, reflecting superior organizational frameworks, multidisciplinary approaches, and advanced therapeutic options. In contrast, nonacademic settings exhibited systemic challenges, including longer delays and limited access to specialized care, contributing to heightened anxiety and lower satisfaction. Demographic differences were also evident, with younger patients and women reporting greater emotional distress, anxiety, and financial fears. These findings underline the need for targeted interventions, such as psychological support and financial counseling, particularly for vulnerable populations. Treatment delays emerged as a pivotal factor influencing satisfaction, with each additional month significantly reducing patient trust and perceived care quality. The data underscore the necessity for systemic reforms to streamline oncology care pathways, particularly in nonacademic settings, to ensure equitable and efficient treatment delivery. Taken together, this research underscores the importance of empathy, communication, and systemic efficiency in fostering a patient-centered approach to oncology care. Addressing the identified gaps through policy and practice changes could significantly enhance patient experiences and outcomes across diverse healthcare settings.

## Supplementary Material

oyag046_Supplementary_Data

## Data Availability

The datasets generated and analyzed during the current study are available from the corresponding author upon reasonable request.
