# Supplementary material for: Empathy, access, and delays: determinants of oncology care satisfaction in Polish academic and nonacademic hospitals
Source: Oncologist. 2026 Aug 7;31(9):oyag046. doi: 10.1093/oncolo/oyag046 (PMC13451092; doi:10.1093/oncolo/oyag046)
Supplement: Supplementary file 1 [file oyag046_supplementary_data.zip › Supplementary_Material_1_Survey_Instrument.docx]

# Supplementary Material 1

Supplementary Material 1. Survey Instrument Used to Assess Patient Satisfaction with Oncology Care in Polish Hospitals.

This supplementary file presents the full English version of the structured questionnaire used in the study assessing patient satisfaction with oncology care in Polish hospitals. The survey was administered anonymously and was designed to capture sociodemographic characteristics, clinical context, patient satisfaction with care organisation and quality, psychological distress, communication quality, and access to oncological services.

## Section I. Sociodemographic and Clinical Characteristics

- Age: <30 years; 30–50 years; 51–70 years; >70 years
- Gender: Female; Male
- Education level: Primary; Secondary; Higher
- Nationality: Polish; Other
- Place of oncology treatment (hospital name)
- Type of hospital: Academic hospital; Other
- Duration of oncological treatment or diagnostic process: <3 months; 3–6 months; 6–12 months; >12 months
- History of surgical treatment prior to systemic therapy: Yes; No; Do not know
- Time since cancer diagnosis: <3 months; 3–6 months; 6–12 months; 1–5 years; >5 years

## Section II. Satisfaction with Treatment Organisation and Care Quality

- Overall satisfaction with the course of treatment
- Satisfaction with availability of medical appointments
- Satisfaction with the quality of medical care received
- Time from diagnosis to initiation of treatment (in months)
- Anxiety related to waiting time before treatment initiation
- Problems with treatment organisation (e.g., long waiting times, limited diagnostic access)
- Perceived adequacy of access to oncology specialists
- Perceived respect and empathy demonstrated by medical staff
- Presence of fear or anxiety prior to treatment initiation

## Section III. Treatment-Related Fear and Anxiety

- Fear related to treatment side effects
- Fear of death
- Fear of pain
- Fear of loss of employment, income, or financial security

## Section IV. Communication, Psychosocial Aspects, and Trust

- Presence of family or friends during medical visits
- Sense of loneliness during treatment
- Receipt of detailed information regarding treatment from physicians
- Adequacy of medical staff availability
- Perceived quality of care provided by medical staff
- Presence of chronic pain during treatment
- Loss of hope regarding recovery
- Access to alternative treatment options and freedom of choice
- Access to innovative oncological therapies (drug programmes, clinical trials)
- Adequacy of time provided by medical staff to answer patient questions
- Comfort during conversations with physicians
- Comfort during conversations with nurses (including 'not applicable' option)
- Deterioration of mental health prompting consideration of psychological support
- Fear of disease progression
- Clarity and comprehensibility of medical explanations
- Independent search for treatment-related information on the internet
- Seeking additional medical consultation outside the treating physician
- Use of non-prescription medications or dietary supplements
- Use of alternative or non-conventional medicine
- Trust in the physician regarding treatment decisions
- Optional open-ended comments on oncology care
