# Supplementary material for: Empathy, access, and delays: determinants of oncology care satisfaction in Polish academic and nonacademic hospitals
Source: Oncologist. 2026 Aug 7;31(9):oyag046. doi: 10.1093/oncolo/oyag046 (PMC13451092; doi:10.1093/oncolo/oyag046)
Supplement: Supplementary file 1 [file oyag046_supplementary_data.zip › Supplemental Table S1.docx]

Supplementary Table S1. Patients characteristics

| **Category** | **Subcategory** | **n** | **Percentage (%)** |
| --- | --- | --- | --- |
| **Gender** | Female | 370 | 67.77 |
|  | Male | 176 | 32.23 |
| **Education** | High School | 260 | 47.62 |
|  | Higher Education | 249 | 45.60 |
|  | Primary Education | 37 | 6.78 |
| **Treatment Location** | Academic Hospital | 296 | 54,21 |
|  | Non-Academic Hospital | 250 | 45,79 |
| **Treatment Duration** | More than 12 months | 261 | 47,80 |
|  | 6-12 months | 136 | 24,91 |
|  | 3-6 months | 98 | 18,13 |
|  | Less than 3 months | 50 | 9,16 |
| **Prior Surgery** | No | 291 | 53,30 |
|  | Yes | 249 | 45,60 |
|  | Don't Know | 3 | 0,55 |
| **Diagnosis Time** | 1 to 5 years ago | 252 | 46,15 |
|  | 6 to 12 months ago | 142 | 26,00 |
|  | 3 to 6 months ago | 97 | 17,77 |
|  | Less than 3 months ago | 35 | 6,41 |
|  | More than 5 years ago | 20 | 3,66 |
