# Supplementary material for: Empathy, access, and delays: determinants of oncology care satisfaction in Polish academic and nonacademic hospitals
Source: Oncologist. 2026 Aug 7;31(9):oyag046. doi: 10.1093/oncolo/oyag046 (PMC13451092; doi:10.1093/oncolo/oyag046)
Supplement: Supplementary file 1 [file oyag046_supplementary_data.zip › Supplementary Figure S1.docx]

**Supplementary Figure S1:** Distribution of fear and satisfaction factors among patients in academic and non-academic hospitals; (A) Distribution of fear and anxiety factors among patients in academic and non-academic hospitals; (B) Distribution of satisfaction factors among patients in academic and non-academic hospitals.
